# Automatic Human Embryo Volume Measurement in First Trimester Ultrasound From the Rotterdam Periconception Cohort: Quantitative and Qualitative Evaluation of Artificial Intelligence

**DOI:** 10.2196/60887

**Published:** 2025-03-31

**Authors:** Wietske A P Bastiaansen, Stefan Klein, Batoul Hojeij, Eleonora Rubini, Anton H J Koning, Wiro Niessen, Régine P M Steegers-Theunissen, Melek Rousian

**Affiliations:** 1 Department of Obstetrics and Gynecology Erasmus MC, University Medical Center Rotterdam The Netherlands; 2 Department of Radiology and Nuclear Medicine Biomedical Imaging Group Rotterdam University Medical Center, Erasmus MC Rotterdam The Netherlands; 3 Department of Pathology Erasmus MC, University Medical Center Rotterdam The Netherlands; 4 University Medical Center Groningen Groningen The Netherlands

**Keywords:** first trimester, artificial intelligence, embryo, ultrasound, biometry, US, Rotterdam, The Netherlands, Cohort, quantitative, qualitative, evaluation, noninvasive, pregnancy, embryonic growth, algorithm, embryonic volume, monitoring, development

## Abstract

**Background:**

Noninvasive volumetric measurements during the first trimester of pregnancy provide unique insight into human embryonic growth and development. However, current methods, such as semiautomatic (eg, virtual reality [VR]) or manual segmentation (eg, VOCAL) are not used in routine care due to their time-consuming nature, requirement for specialized training, and introduction of inter- and intrarater variability.

**Objective:**

This study aimed to address the challenges of manual and semiautomatic measurements, our objective is to develop an automatic artificial intelligence (AI) algorithm to segment the region of interest and measure embryonic volume (EV) and head volume (HV) during the first trimester of pregnancy.

**Methods:**

We used 3D ultrasound datasets from the Rotterdam Periconception Cohort, collected between 7 and 11 weeks of gestational age. We measured the EV in gestational weeks 7, 9 and 11, and the HV in weeks 9 and 11. To develop the AI algorithms for measuring EV and HV, we used nnU-net, a state-of-the-art segmentation algorithm that is publicly available. We tested the algorithms on 164 (EV) and 92 (HV) datasets, both acquired before 2020. The AI algorithm’s generalization to data acquired in the future was evaluated by testing on 116 (EV) and 58 (HV) datasets from 2020. The performance of the model was assessed using the intraclass correlation coefficient (ICC) between the volume obtained using AI and using VR. In addition, 2 experts qualitatively rated both VR and AI segmentations for the EV and HV.

**Results:**

We found that segmentation of both the EV and HV using AI took around a minute additionally, rating took another minute, hence in total, volume measurement took 2 minutes per ultrasound dataset, while experienced raters needed 5-10 minutes using a VR tool. For both the EV and HV, we found an ICC of 0.998 on the test set acquired before 2020 and an ICC of 0.996 (EV) and 0.997 (HV) for data acquired in 2020. During qualitative rating for the EV, a comparable proportion (AI: 42%, VR: 38%) were rated as excellent; however, we found that major errors were more common with the AI algorithm, as it more frequently missed limbs. For the HV, the AI segmentations were rated as excellent in 79% of cases, compared with only 17% for VR.

**Conclusions:**

We developed 2 fully automatic AI algorithms to accurately measure the EV and HV in the first trimester on 3D ultrasound data. In depth qualitative analysis revealed that the quality of the measurement for AI and VR were similar. Since automatic volumetric assessment now only takes a couple of minutes, the use of these measurements in pregnancy for monitoring growth and development during this crucial period, becomes feasible, which may lead to better screening, diagnostics, and treatment of developmental disorders in pregnancy.

## Introduction

The current standard for monitoring growth and development during early pregnancy is the crown-rump length (CRL). Early measurements of the CRL are used in standard clinical practice to estimate gestational age. Moreover, CRL measurements can be used to predict miscarriages and are associated with estimated fetal weight, birth weight, and adverse pregnancy outcomes [1-5].

Volumetric measurements of the human embryo during early pregnancy are a novel way to assess growth and development. Conventional manual tracing methods, such as Virtual Organ Computer Added analysis (VOCAL), estimate the volume by drawing contours around the embryo in rotational steps, without including the limbs, resulting in an underestimation of the volume [6,7]. More recently, within the Erasmus MC, University Medical Center, in Rotterdam, the Netherlands, a Virtual Reality (VR) system has been developed to visualize the 3D ultrasound images and perform volumetric measurements [8,9]. Measuring the embryonic volume (EV) and embryonic head volume (HV) using VR is reliable and reproducible [10,11]. Reliable and reproducible measurements are crucial for clinical implementation, especially since several studies have shown that the EV offers a better assessment of embryonic growth than the CRL [8,12]. In addition, EV has also been associated with miscarriage [2], small-for-gestational age (GA; birth weight <10th percentile) [5], folic acid supplement use, maternal smoking and inadequate fruit intake [13], maternal vitamin B_12_ and homocysteine concentrations [14,15], and maternal social and medical risk factors [16]. On top of these associations, in the case of structural congenital anomalies and aneuploidy, the EV showed earlier signs of deviation, by being significantly smaller, in contrast to CRL, which was within the normal range [17,18]. HV has been associated with maternal age, periconceptional smoking, conception via in vitro fertilization (IVF) or intracytoplasmic sperm injection (ICSI) [11]. Hence, volumetric measurements during the first trimester provide a valuable screening and diagnostic tool for early detection of adverse birth outcomes, congenital anomalies, and aneuploidy. By enabling timely interventions and preventive measures, these measurements complement conventional biometry and can improve pregnancy outcomes and the lifelong health of the developing fetus.

Measuring the EV takes from 5 to 10 minutes for an experienced rater and is thus too time-consuming for implementation in daily clinical practice [10]. Moreover, measuring volume semiautomatically using VR, or manual using tracing methods (eg, VOCAL) requires specific training and may lead to inter- and intrarater measurement variability [8,19].

**Figure 1:**
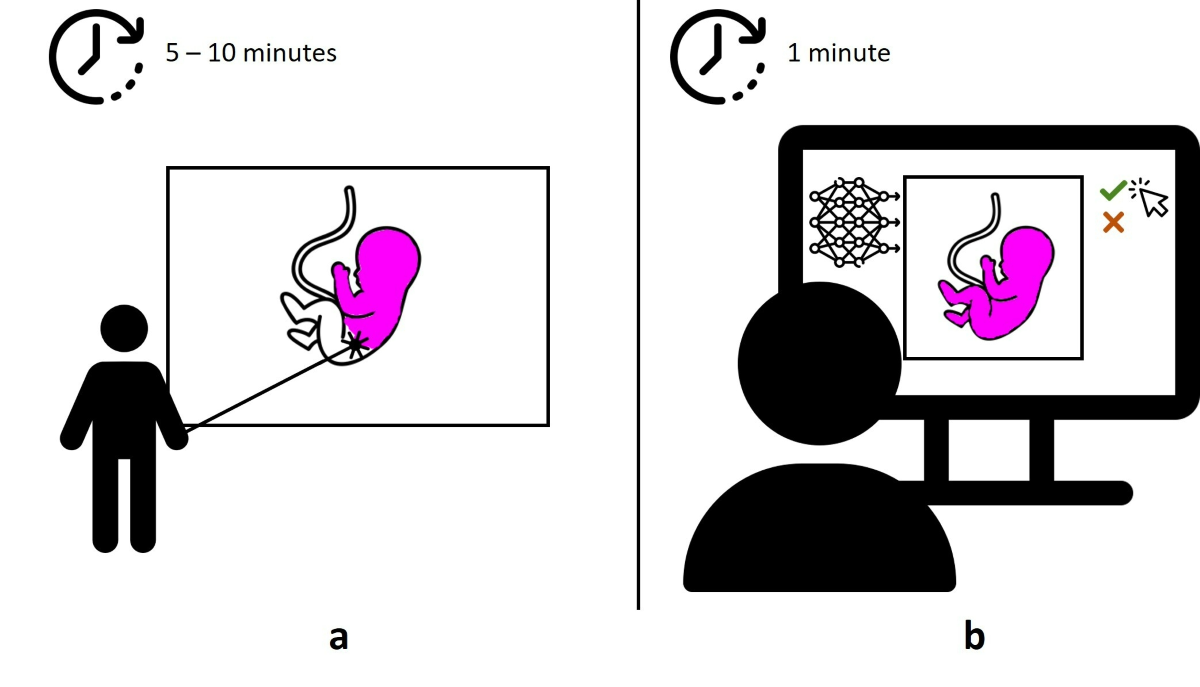
Schematic illustration of virtual reality and artificial intelligence measurements. (A) Virtual reality measurements, where the operator manually identifies specific embryonic regions within the image. (B) Illustration of artificial intelligence measurements, where the operator only visually has to inspect the results. In this study, all reported results were recorded prior to quality checking.

To enable clinical implementation of volumetric measurement in a time-saving and reliable manner, we propose a fully automatic artificial intelligence (AI) method to segment (delineate) the complete embryo, starting at 7 weeks GA, and the embryonic head, starting at 9 weeks GA. The key difference between VR and AI, highlighted in Figure 1, is that VR measurements are semiautomatically, whereas AI measurements are fully automatic, eliminating the need for any interaction or specialized expertise to obtain the measurements beyond quality checking. In the literature, 3 other automatic AI-based methods for segmentation of the embryo have been published [20-22]. However, in these methods, the algorithms were tailored for data acquired starting at 10 weeks GA, and are therefore not directly applicable to measurements starting at 7 weeks GA. Only Ryou et al [21] segmented the head subsequently to the segmentation of the embryonic volume. Hence, we are the first to address automating volumetric measurements of the human embryo and embryonic head, highlighting the novelty of our study.

In this study, we propose the use of nnU-Net, a state-of-the-art publicly available segmentation method [23], to automatically measure the EV and HV. nnU-Net has excelled in medical image segmentation, achieving top performance with minimal manual tuning across tasks like brain and liver tumor segmentation (medical segmentation decathlon), abdominal organ segmentation, and cardiac magnetic resonance imaging segmentation [23]. For the embryo and fetus, so far published studies successfully segmented brain structures [24], lungs, and liver [25] in magnetic resonance imaging and cardiac structures in echocardiography [26], both in the second and third trimester. Given this success, we hypothesize that nnU-net can also be successful in segmentation of the embryo and embryonic head in first trimester ultrasonography.

Our aim was to evaluate the accuracy and reliability of this AI-based approach compared with the already validated VR segmentation method. We assessed performance through both quantitative and qualitative analyses. Quantitatively, we compared volume differences between AI and VR segmentations and examined factors influencing segmentation accuracy. Qualitatively, expert raters visually evaluated the segmentations using a newly developed protocol, which also enabled us to estimate the time-saving potential of the AI approach. This dual evaluation highlights the novelty of our work, offering insights into the reliability and efficiency of AI-based segmentation in clinical workflows.

## Methods

### Data

The data used for this study was collected within the Rotterdam Periconception Cohort (Predict Study). The Rotterdam Periconception Cohort is an ongoing prospective tertiary hospital–based cohort conducted since November 2010 at the Department of Obstetrics and Gynecology of the Erasmus MC, University Medical Center, Rotterdam, The Netherlands [27,28]. The included women were at least 18 years old, with an ongoing singleton pregnancy of less than 10 weeks GA.

The participating women received transvaginal 3D ultrasound scans in gestational weeks 7, 9, and 11. Trained sonographers performed the ultrasound examinations using a 6-12 MHz transvaginal probe with GE Voluson E8 equipment (GE, Zipf Austria). The scans were stored as Cartesian volumes using specialized software for 3D ultrasound (4D View, GE Medical Systems). No additional preprocessing, such as region-of-interest selection or noise filtering, was performed*.*

### Measurements Using VR

The volume measurements in VR were performed using a semiautomatic region-growing segmentation algorithm [5]. To perform VR measurements, either a fully immersive VR-room or desktop VR system is needed. The interactive VR visualization of the ultrasound image can be manipulated using a virtual pointer controlled by a wireless joystick. The joystick enables voxel inclusion or exclusion to perform segmentation (Figure 1A). The algorithm is semiautomatic, since after manual selection of the seed point for region growing a first rough segmentation of the embryo is created, which subsequently is refined by manual in- and exclusion of voxels using the virtual pointer. The threshold used for region growing was calibrated using a phantom with a known volume [29]. EV encompasses the entire embryo and was mainly measured in gestational weeks 7, 9, and 11. EV measurements were followed by HV measurements, where the embryonic head was isolated using a cutting plane between the lowest chin point and the fourth ventricle’s lowest point in the midsagittal plane [11]. The accuracy and reliability of both measurements were shown to be excellent [10,11]. The measurements time for the EV ranges between 5 and 10 minutes [10]. HV is measured after the EV and therefore takes even longer. Finally, the quality of each ultrasound images for EV measurement was rated as excellent, good, or moderate. Rating was based on the following criteria: (1) Excellent: high resolution image, little to no noise, embryo mostly surrounded by amniotic fluid (>75% in midsagittal view), brain ventricles and structures clearly visible. (2) Good: moderate resolution image, little to moderate noise, embryo partly lies against uterine wall with clear edges, brain ventricles visible. (3) Moderate: low resolution image, noisy, shadowing but occluded body parts can be estimated from surrounding voxels, embryo lies against uterine wall with blurry edges, fourth ventricle visible.

**Figure 2:**
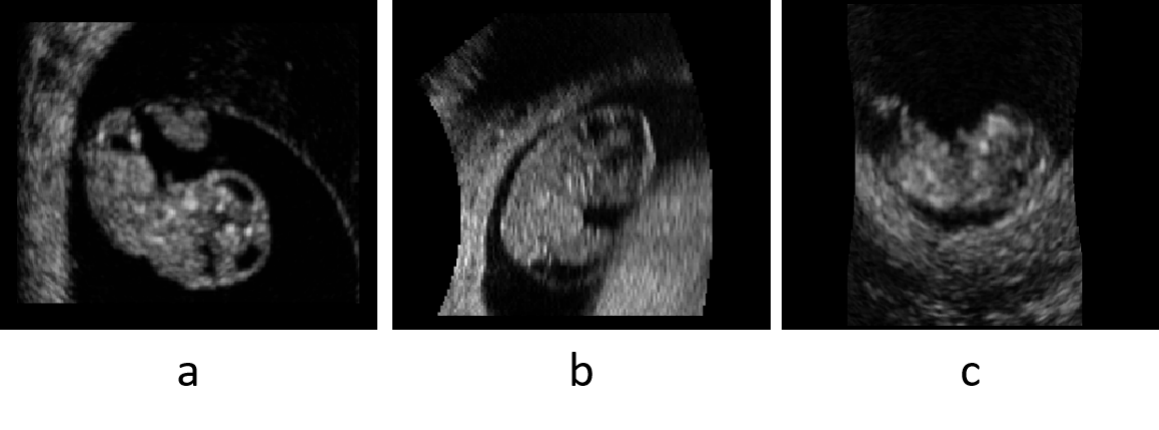
Embryo at 9 weeks gestational age for different quality ratings in midsagittal view. (A) Excellent image quality: high resolution, embryo surrounded by amniotic fluid, and clear brain ventricles. (B) Good image quality: moderate resolution, moderate noise, embryo touches uterine wall with clear boundaries, and brain ventricles visible. (C) Low resolution: noisy image, blurry edge between embryo and uterine wall, and fourth ventricle visible.

Figure 2 shows examples of images of an embryo at 9 weeks gestational age for different quality ratings in midsagittal view.

### Measurements Using AI

We used nnU-net to measure the EV and HV, which is a state-of-the-art publicly available segmentation method based on deep learning [23]. nnU-net configures itself automatically based on the available imaging data. The algorithm takes as input the 3D ultrasound image and outputs the corresponding predicted segmentation. During development, the algorithm learned to set its internal parameters by minimizing the difference between the predicted segmentation and the segmentations obtained in VR. Two separate models were developed: one for segmenting the embryo and another for the embryonic head. We used nnU-net version 1.6.5 (Isensee) with default settings on a Nvidia A40 48GB GPU with an AMD EPYC 7742 CPU using 20 GB of RAM. Once the algorithm is trained application to new data becomes fully automatic. Multimedia Appendix 1 provides details on the configuration of nnU-net.

### Dataset Characteristics

We included participants for which the segmentation of volume measurement performed in VR was saved. For the EV, this led to a dataset consisting of 1016 ultrasound images of 539 participants. The ultrasound data was divided into three subsets: (1) development set, (2) test set, and (3) future test set. The development set contained 648 ultrasound scans of 341 participants acquired before 2020 and was used to develop the AI model. The test set contained 163 ultrasound scans of 82 participants acquired before 2020 and was used to evaluate the model’s performance on data not used during development. The future test set contained 204 ultrasound scans of 116 participants acquired in 2020, which was used to investigate how well the model generalizes to data acquired later in time. Details of these 3 datasets are provided in Table 1; using the chi-Squared test we evaluated whether the distributions of dataset characteristics were comparable among the subsets. Although some ultrasound images were not acquired in weeks 7, 9, or 11, we included them in the study. GA in spontaneous pregnancies was calculated from the first day of the last menstrual period. GA in pregnancies achieved using IVF or ICSI after fresh embryo transfer was calculated from the oocyte retrieval day plus 14 days, in case of IVF or ICSI after frozen-thawed embryo transfer, GA was calculated from the oocyte retrieval day plus 19 days. In case of a cryopreserved embryo transfer, the calculation depended on the number of days between oocyte retrieval and embryonic cryopreservation.

**Table 1:** Data characteristics of the 3 datasets for the embryonic volume and head volume measurement. *P* values for the chi-square test are given.

		Development					Test					Future test				*P* value
**EV^a^**																
	Participants, n		341		82						116					—^b^
	Ultrasound scans, n (%)		648 (100)		163 (100)						204 (100)					—
**GA^c^ (%)**																
	Week 7		141 (21.8)		40 (25)						28 (13.9)					.20
	Week 9		248 (38.3)		67 (40.9)						73 (36.1)					.86
	Week 11		184 (28.4)		44 (26.8)						81 (40.9)					.16
	Other		75 (11.5)		12 (7.3)						22 (9.1)					—
**Outcome**																
	No adverse outcome			582 (89.8)			151 (92.1)				196 (97)					.87
	Adverse outcome			66 (10.2)			12 (7.9)				6 (3)					.15
**BMI (%)**																
	Not obese (<30)			553 (85.8)			141 (86))				146 (72,2)					<.001
	Obese (≥30)			93 (14.4)			23 (14)				29 (14.4)					>.99
	Missing			2 (0.2)			0 (0)				29 (13.4)					—
**Ultrasound quality (%)**																
	Excellent			200 (30.9)			59 (36)				78 (38.6)					.65
	Good			254 (39.2)			53 (32.3)				55 (27.2)					.33
	Moderate		179 (27.6)			49 (30)				64 (31.7)					.87	
	Missing		15 (2.3)			3 (1.7)				4 (2.5)					—	
**HV^d^**																
	Participants, n	261			65						58					—
	Ultrasound scans, n (%)	383 (100)			92 (100)						80 (100)					—
**GA (%)**																
	Week 9		185 (48.3)			43 (46.7)				37 (46.3)					.98	
	Week 11		175 (45.7)			44 (47.8)				39 (48.8)					.95	
	Other		23 (6)			5 (5.5)				4 (4.9)					.95	
**Outcome (%)**																
	No adverse outcome	381 (99.4)			92 (100.0)			80 (100)					>.99			
	Adverse outcome	2 (0.6)			0 (0)			0 (0)					.55			
**BMI (%)**																
	Not obese (<30)	326 (85.1)			78 (84,8)				64 (80)					.91		
	Obese (≥30)	57 (14.9)			14 (15.2)				8 (10)					.53		
	Missing	0 (0)			0 (0)				8 (10)					—		

^a^EV: embryonic volume.

^b^Not applicable.

^c^GA: gestational age.

^d^HV: head volume.

For the HV, a total of 475 ultrasound scans of 326 participants were included. The data were divided over similar subsets. The development set contained 383 ultrasound scans of 261 participants acquired before 2020. The test set contained 92 ultrasound scans of 65 participants acquired before 2020. The future test set contained 80 ultrasound scans of 58 participants acquired in 2020. The characteristics of these 3 datasets can be found in Table 1.

### Quantitative Analysis

We evaluated the AI algorithm’s performance for EV and HV quantitatively, comparing AI and VR segmentations in the test and future test sets. We reported the intraclass correlation coefficient (ICC), Dice score, absolute volume error, and absolute relative volume error. ICC measures interobserver reliability, and a score of 0.90 or higher indicates good agreement. The dice score indicates segmentation overlap, with a score of 1 indicating perfect overlap and 0 indicating no overlap [30]. Absolute volume error is the difference between AI and VR volumes, while absolute relative volume error is absolute volume error divided by the volume obtained in VR.

For the Dice score and volume errors, we report the mean and SD over the test and future test set. We used Bland-Altman plots to assess agreement based on the relative volume error [31]. We also investigated the factors that may influence the model’s performance: GA, adverse outcomes (miscarriage, termination of pregnancy, intrauterine fetal death, stillbirth, postpartum death, and congenital malformations identified at birth), maternal BMI, image quality, and the number of available training samples. This was done by statistical comparison of the evaluation metrics for: datasets within a gestational age versus the rest, participants with adverse outcome versus no adverse outcome, participants with maternal obesity versus not obesity, datasets with excellent ultrasound quality versus good quality, and moderate quality versus good quality. For HV image quality was not available, and the influence of pregnancy outcome was not tested since adverse outcomes were not present in the test set. To investigate the influence of the number of available samples for development, we created smaller development sets while preserving the ratios of the characteristics given in Table 1. Details on the subsets can be found Table S2.1 in Multimedia Appendix 2.

Finally, we evaluated the precision of the AI measurements compared with the VR measurements. To this end, we analyzed the association between birth weight (exposure) and longitudinal embryonic volumetric measurements (outcome) using a linear mixed model. As a reference, also the association with CRL was analyzed, which was found to be significant in previous research [3]. This analysis was performed within the test set and future test set. CRL measurements were repeated 3 times, and the average of these measurements was used [10]. The birth weight was retrieved from medical records and *z* scores were calculated based on Dutch reference growth curves adjusted for GA and fetal sex [32].

### Qualitative Analysis

Experienced raters conducted a qualitative analysis of the segmentations through visual inspection. All raters followed standardized training to perform these measurements [28]. The EV was rated by MR (gynecologist, > 10 years of experience in VR measurements, rater (1) and BH (biomedical Ph.D. student, >3 years of experience in VR measurements, rater (2). The HV was rated by MR and ER (biomedical Ph.D. student, >3 years of experience in VR measurements, rater (3). A total of 60 segmentations for the EV and 40 for the HV were analyzed. The ultrasound images were selected randomly for the future test set but were evenly distributed over gestational weeks and image quality. The HV images were selected from gestational weeks 9 and 11. All selected images were from unique participants.

Each rater performed 2 rounds, with half of the segmentations obtained using AI within each round. In addition, rater 1 performed the first round twice to assess intrarater reliability. For each image, the segmentation shown in the first round, AI or VR, was determined at random. The images were always shown in the same order, and rounds or repetitions were conducted at least 2 weeks apart to prevent a recall bias. During the experiment, the raters were blinded to the method used, their rating in the other round, and the results of the other rater.

The rating was performed using the same VR set-up as for acquiring the VR segmentations. The following score was assigned: (1) Excellent: Only voxels part of the embryo or embryonic head are included in the segmentation, segmentation is complete. (2) Minor adjustments required: Undersegmentation: excluding voxels part of embryo or embryonic head, oversegmentation: including voxels part of amniotic fluid, yolk sac, placenta, or uterine wall, holes within the interior, and exclusion of part of the limbs. (3) Major adjustments required: Limbs not included in the segmentation, estimated cutting plane for HV superior (undersegmentation) over inferior (oversegmentation).

For scores 2 and 3 a description was noted of the necessary adjustment. Finally, the time needed to rate the segmentations was noted as well. We calculated Cohen to evaluate the agreement among raters [33]. Finally, all given scores were summarized in confusion matrices, which is a table showing the scores of both raters.

### Statistical Analysis

Data characteristics were compared using the chi-squared test. The Mann-Whitney *U* test was used to compare unpaired data, and the Wilcoxon signed-rank test was used for paired data. *P* values of <.05 were considered significant. Absolute volume error was not statistically analyzed due to positive correlation with GA. The association between birth weight and first trimester longitudinal CRL and EV measurements was studied using linear mixed models. With a linear mixed model, repeated measurements of the same participant can be included, by including the GA as time descriptor. To model the individual growth trajectories, a random intercept term was used, and the EV was log transformed to linearize the relationship between GA and EV (Figure S3.1 in Multimedia Appendix 3). All analyses were performed using Python 3.7 (Python Software Foundation).

### Ethical Considerations

This study was approved by the local medical ethical and institutional review board of the Erasmus MC, University Medical Center, Rotterdam, The Netherlands (MEC-2004-227). Prior to participation, all participants provided written informed consent.

## Results

### Quantitative Analysis

**Figure 3:**
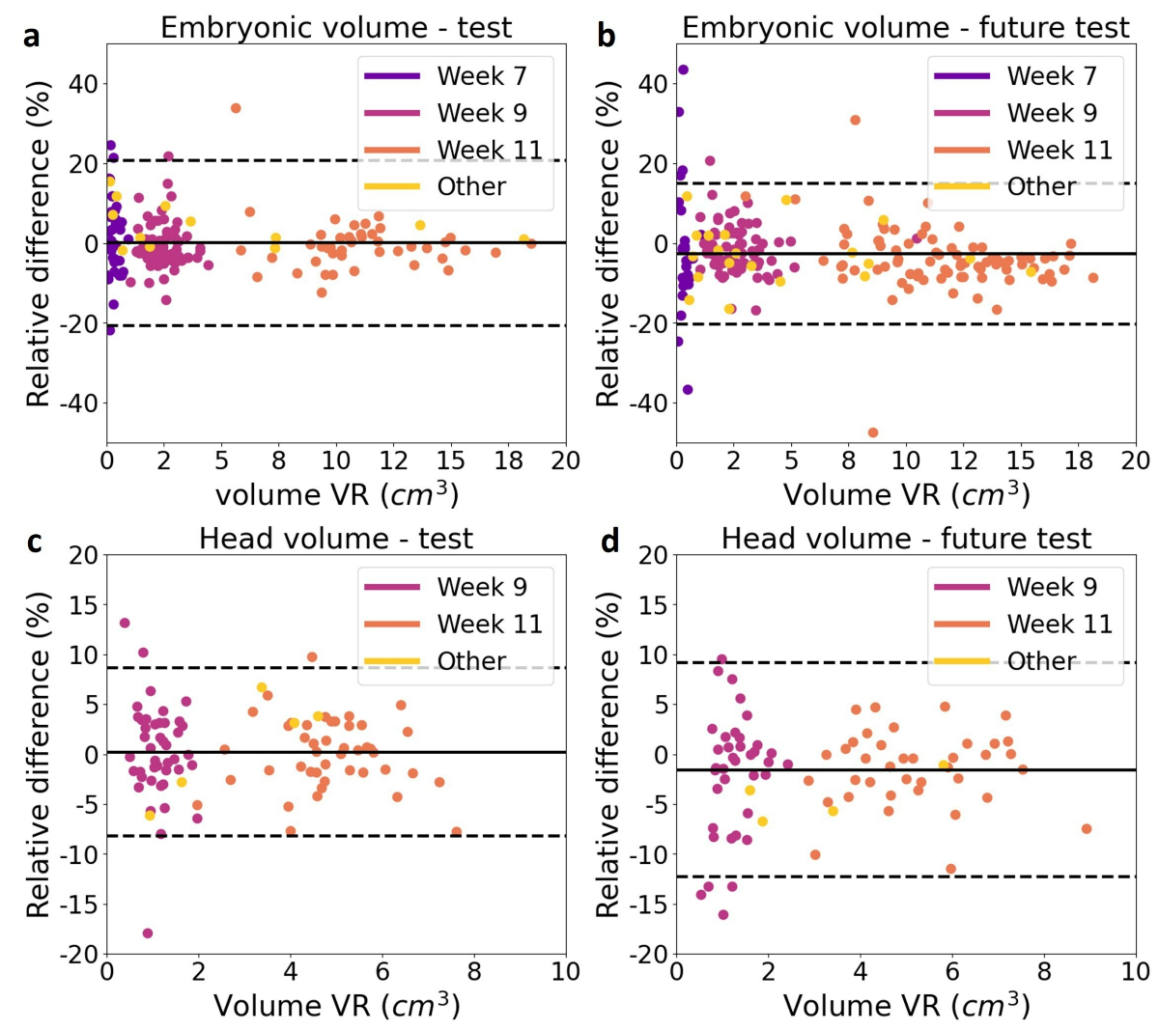
Bland-Altman plots for the embryonic volume (EV) and head volume (HV). The relative difference between the volume obtained using virtual reality (VR) and using artificial intelligence (AI) was plotted against the volume obtained using VR. The solid line represents the mean relative difference and the dotted lines represent the limits of agreement (mean relative difference SD 1.96). The colors indicate the week GA. (A) EV for the test set, (B) EV for the future test set, (C) HV for the test set, and (D) HV for the future test set.

We found an average runtime of 57 seconds for the EV and 41 seconds for the HV. We found excellent agreement between the volume measured using AI and VR, for both the test set and future test set: for the EV we obtained respectively an ICC of 0.998 and 0.996, and for the HV 0.998 and 0.997. However, when comparing the Dice score and absolute relative volume error for the test set and future test set in Tables 2 and 3, we found small, but statistically significant, differences. The Bland-Altman plots for both EV and HV in Figure 3 explain these significant differences: the mean relative differences were respectively for test and future test set 0.01% and –2.74% for the EV and 0.22% and –1.56% for the HV. Hence, on the future test set, the models slightly underestimated the volume compared with the test set.

**Table 2:** Quantitative performance measures for the embryonic volume.

		Number	Dice score, mean (SD)	*P* value Dice score	Absolute volume error (cm^3^), mean (SD)	Absolute relative volume error (%), mean (SD)	*P* value absolute relative volume error
Test set		163	0.941 (0.044)	—^a^	0.160 (0.296)	4.99 (5.13)	—
**GA^b^**							
	Week 7	40	0.907 (0.060)	<.001	0.021 (0.015)	7.51 (5.78)	<.001
	Week 9	67	0.954 (0.022)	.002	0.099 (0.102)	4.05 (3.96)	.03
	Week 11	44	0.954 (0.028)	.005	0.389 (0.362)	4.10 (5.35)	.03
	Other	12	0.931 (0.050)	—	0.132 (0.169)	5.12 (4.69)	—
**Outcome**							
	No adverse outcome	151	0.943 (0.04)	—	0.167 (0.258)	4.77 (4.99)	—
	Adverse outcome	12	0.912 (0.067)	.04	0.079 (0.062)	7.70 (5.77)	.005
**BMI**							
	Not obese (<30)	140	0.941 (0.045)	—	0.166 (0.256)	4.86 (4.90)	—
	Obese (≥30)	23	0.940 (0.031)	.13	0.126 (0.200)	5.80 (6.18)	.05
**Ultrasound quality**							
	Excellent	59	0.960 (0.023)	<.001	0.114 (0.145)	3.89 (3.92)	.002
	Good	53	0.947 (0.027)	—	0.120 (0.155)	3.98 (4.24)	—
	Moderate	48	0.911 (0.059)	<.001	0.267 (0.378)	7.28 (6.32)	<.001
Future test set		204	0.935 (0.041)	.004	0.370 (0.574)	6.62 (6.69)	<.001

^a^Not applicable.

^b^GA: gestational age.

**Table 3:** Quantitative performance measures for the head volume.

			Number		Dice score, mean (SD)		*P* value dice score		Absolute volume error (cm^3^), mean (SD)		Absolute relative volume error, mean (SD)		*P* value absolute relative volume error
Test set			92		0.955 (0.016)		—^a^		0.088 (0.131)		3.23 (2.85)		—
**GA^b^**													
	Week 9	43		0.952 (0.018)		.06		0.036 (0.033)		3.52 (3.43)		.40	
	Week 11	44		0.960 (0.013)		.09		0.135 (0.113)		2.80 (2.12)		.14	
	Other	5		0.960 (0.014)		—		0.127 (0.069)		4.53 (1.61)		—	
**BMI**													
	Not obese (<30)	78		0.955 (0.016)		—		0.085 (0.091)		3.04 (2.65)		—	
	Obese (≥30)	14		0.954 (0.016)		.42		0.107 (0.121)		4.34 (3.50)		.12	
Future test set			80		0.946 (0.019)		.001		0.108 (0.167)		3.96 (4.13)		.49

^a^Not applicable.

^b^GA: gestational age.

We statistically compared the performance measures between the test set and the future test set and for the test set: every gestational week group separately to the rest, adverse outcome to no adverse outcome, obese to not obese, excellent ultrasound quality to good quality, and moderate quality to good quality.

We statistically compared the performance measures between the test set and the future test set, and for the test set: every gestational week group separately to the rest, and obese to not obese.

**Figure 4:**
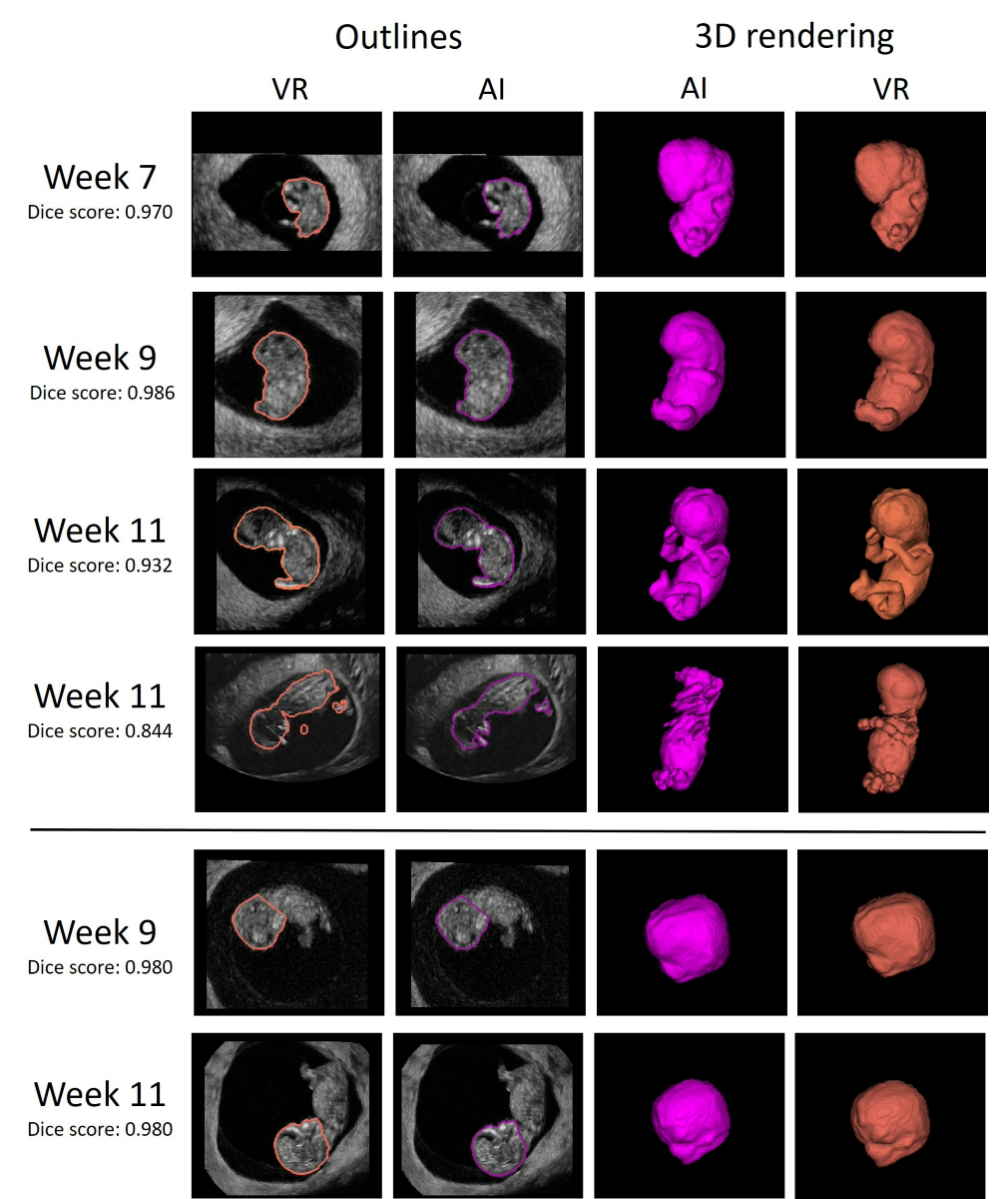
Visualization of the segmentations obtained using artificial intelligence (AI) and virtual reality (VR). For both the AI and VR segmentations on the left the outline is given, and on the right the 3D rending. Rows 1-3 show successful examples for embryonic volume (EV) measurement and column 4 shows an example where the quality of the ultrasound hampered the AI segmentation, caused by shadow near the fetal head. Rows 5 and 6 show successful examples of head volume measurement.

In Figure 4 which shows a visualization of the segmentations, the AI segmentations appear smoother than the VR ones. This can be explained by the fact that, in VR, the segmentations are corrected manually with a spherical brush, which causes the nonsmooth edges. Finally, in the fourth row of Figure 4, an example is shown where part of the head and limbs are missing, caused by low ultrasound image quality.

The data characteristics for development, test, and future test set were only significantly different for the not obese participants in the EV datasets (Table 1), which can be explained by the high number of missing BMI measurements in the future test set. Regarding the influence of GA on the performances, we observed in Table 2 that for gestational week 7 the Dice score and absolute relative volume error were significantly worse, and for weeks 9 and 11 these were significantly better. No significant difference was found between the results for the other surrounding weeks GA and the results for weeks 7, 9, and 11. For pregnancy outcome, the absolute relative volume error was significantly worse in case of an adverse outcome. However, the group with an adverse outcome was relatively small, and 7 out of the 12 images were taken before gestational week 11, which led to worse performances as well. For maternal BMI, we found no significant influence on the performance of the AI algorithm in terms of Dice score. However, for maternal BMI, we found a small, but significant, decrease in performance for the absolute relative volume error. For ultrasound image quality we found, as expected, that excellent quality gives significantly better results, and moderate quality gives significantly worse results. Data characteristics (eg, distribution of image quality per gestational week) in the compared subgroups can be found Table 4.1 in Multimedia Appendix 4.

Similarly, for the HV segmentations, we evaluated the influence of GA and maternal BMI. In Table 3 we observed that maternal BMI has no significant influence on HV measurements. Regarding GA, we observed that images not acquired in weeks 9 and 11 had significantly higher relative volume errors (weeks 8, 10, and 12). Data characteristics (eg, distribution of cases with obesity per gestational week) in the compared subgroups can be found Table 4.2 in Multimedia Appendix 4.

Regarding the number of samples needed for development, in Table 4 we observed for both EV and HV, that the Dice score and absolute relative volume error deteriorated slightly as the number of data samples used decreased. For both measurements, when 40% or less of the development dataset was used, we observed that the results deteriorated significantly. On the smallest development dataset of around 20 ultrasound images, we observed a mean Dice score of 0.888 and 0.946 for the EV and HV respectively, which for the EV led to relative deviations in the volume of around 20%.

**Table 4:** Results for evaluating the influence of the number of samples used for development.

		Dice score, mean (SD)	*P* value	Absolute relative volume error (%), mean (SD)	*P* value
**EV^a^ (%)**					
	100	0.941 (0.044)	—^b^	4.99 (5.13)	—
	80	0.934 (0.086)	.09	5.12 (5.18)	.14
	60	0.934 (0.086)	.33	4.99 (5.04)	.39
	40	0.933 (0.077)	<.001	6.04 (7.40)	.02
	20	0.925 (0.105)	<.001	6.72 (9.30)	<.001
	10	0.922 (0.101)	<.001	7.48 (9.26)	<.001
	5	0.916 (0.120)	<.001	12.8 (64.7)	<.001
	2.5	0.888 (0.109)	<.001	21.4 (133.5)	<.001
**HV^c^ (%)**					
	100	0.955 (0.016)	—	3.23 (2.85)	—
	80	0.955 (0.016)	.21	3.27 (2.77)	.37
	60	0.954 (0.017)	.07	3.38 (2.96)	.59
	40	0.953 (0.017)	<.001	3.36 (2.93)	.32
	20	0.951 (0.018)	<.001	3.62 (3.20)	.04
	10	0.949 (0.019)	<.001	3.94 (3.60)	.02
	5	0.946 (0.019)	<.001	4.13 (3.66)	.006

^a^EV: embryonic volume.

^b^Not applicable.

^c^HV: head volume.

Table 5 shows the association between birth weight and CRL, birth weight and EV obtained using VR, and birth weight and EV obtained using AI. The flowchart of the study population can be found (Figure S3.2 in Multimedia Appendix 3), along with the baseline characteristics (Table S3.4 in Multimedia Appendix 3). For the EV measured using VR and AI the effect estimates () are comparable and the 95% CIs are overlapping, indicating that despite the difference in measurement method, growth trajectories are comparable estimated. All effect estimate () were positive and therefore give the increase of the growth trajectory during the first trimester per unit increase in birth weight (in grams). We found no significant associations between birth weight and CRL or EV.

**Table 5:** Associations between first trimester growth trajectories, quantified by Crown-Rump Length, embryonic volume obtained using virtual reality, and embryonic volume obtained using artificial intelligence, and birthweight^a^.

	β	95% CI	*P* value
CRL^b^	0.304	–0.169 to 0.776	.21
EV^c^ (VR^d^)	0.026	–0.018 to 0.070	.24
EV (AI^e^)	0.027	–0.016 to 0.070	.22

^a^Effect estimate β, *P* value, and the 95% CI are given for a linear mixed model with gestational age as the time predictor.

^b^CRL: crown-rump length.

^c^EV: embryonic volume. The EV was log-transformed to linearize the relationship between GA and EV.

^d^VR: virtual reality.

^e^AI: artificial intelligence.

### Qualitative Analysis

**Figure 5:**
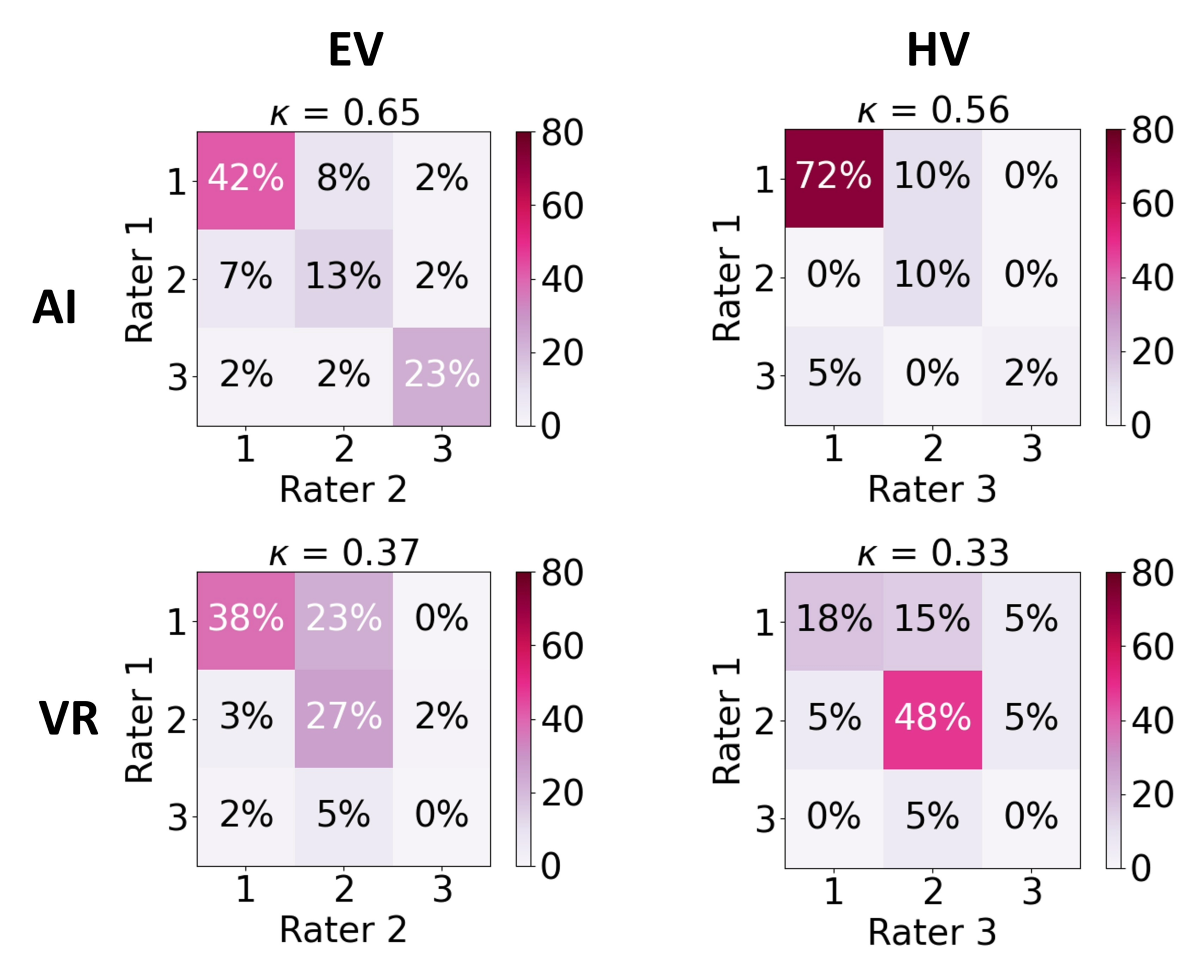
Confusion matrices for the qualitative rating, showing the scores given by both raters. The percentages on the diagonal show where the raters agreed. Score 1 indicates that no adjustments were needed, score 2 indicate that only minor adjustments were needed (filling holes, adding part of limb, under- or oversegmentation at the border), and score 3 indicates that major adjustments were needed (missing limbs and wrongly estimated cutting plane). Cohen κ is indicated by κ, where κ between 0.21 to 0.4 indicates fair agreement and κ between 0.61 to 0.8 substantial agreement.

Qualitative rating of the EV segmentations took on average 45 (SD 12) seconds per image for rater 1 and 1 minute 3 seconds (SD 24 s) for rater 2. Rating of the HV segmentations took on average 42 (SD 7) seconds for rater 1 and 1 minute 6 seconds (SD 20 s) for rater 3. Hence, we found that the total measurement time (run-time algorithm and rating) took on average less than 2 minutes per ultrasound image. Figure 5 shows the confusion matrices for both the EV and HV addressing interrater variability. Figure S5.1 in Multimedia Appendix 5 shows confusion matrices for intrarater variability. For the EV, we found an interrater Cohen κ of 0.65 when using AI (intrarater Cohen κ=0.73), and an interrater Cohen κ of 0.37 when using VR (intrarater Cohen κ=0.47). Similarly, for the HV, we found an interrater Cohen κ of 0.56 when using AI (intrarater Cohen κ=0.71), and an interrater Cohen κ of 0.333 when using VR (intrarater Cohen κ=0.83). For interrater agreement, when both raters agreed, we observed that for EV, no adjustments were needed in 42% of cases with AI compared with 38% with VR. For HV, this percentage was 72% with AI versus 18% with VR. In addition, for EV, 23% of cases required major adjustments with AI, while none did with VR. Conversely, with VR, more minor adjustments (27% vs 13%) were needed. Similar numbers were observed for intrarater agreement.

**Figure 6:**
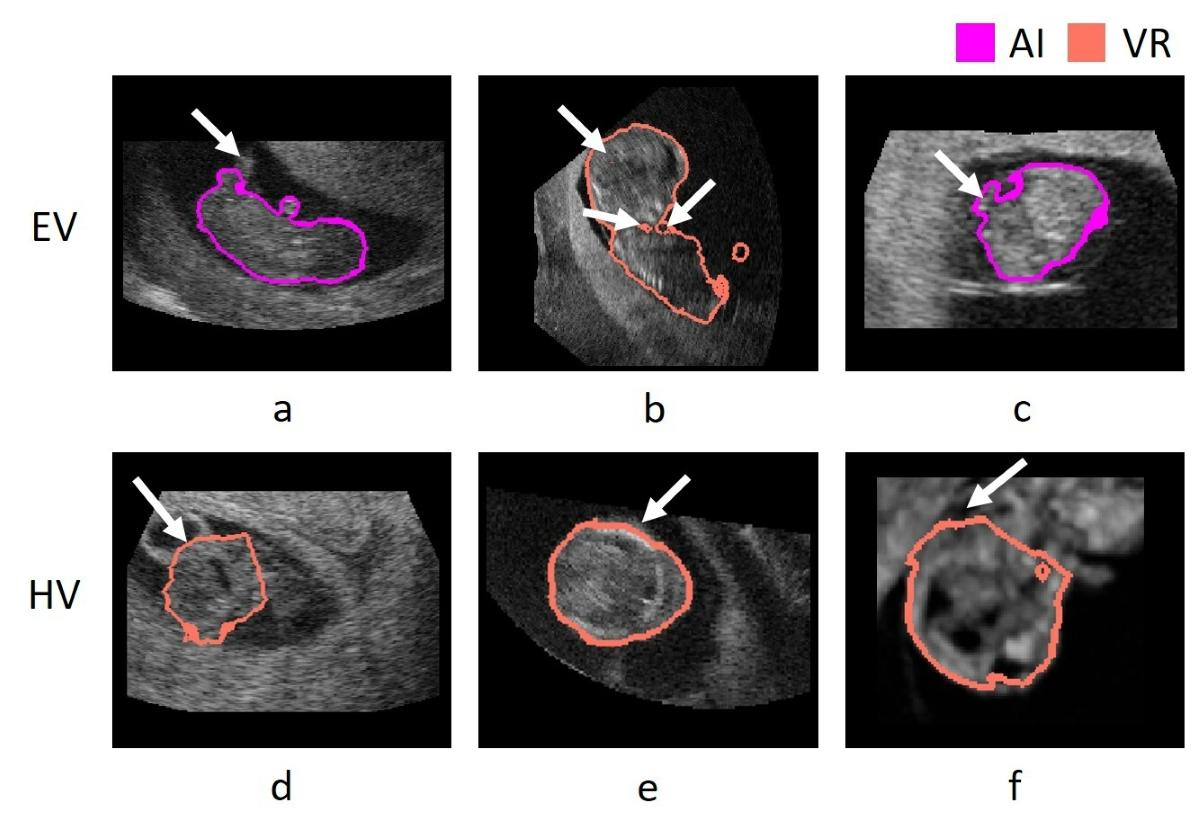
Visualization of minor and major adjustments denoted during the rating of artificial intelligence (AI) and virtual reality (VR) segmentations. (A) AI segmentation at gestational week 9 with part of the leg missing. (B) VR segmentation at gestational week 11 with holes in the segmentation mask. (C) AI segmentation at gestational week 7, due to low image quality, the umbilical cord is partly included in the segmentation. (D) VR segmentation at gestational week 11, where the mask oversegments the head by including part of the yolk sac. (E) VR segmentation at gestational week 11, where the mask undersegments the head. (F) VR segmentation at gestational week 9, where part of the fourth ventricle is missing, which leads to underestimation of the cutting plane.

Table 6 summarizes minor (score 2) and major (score 3) adjustments for EV and HV, which were needed per gestational week by both raters. Using AI, limbs were more frequently missing (AI 32 cases versus VR 15 cases). Missing limbs occurred more frequently in gestational week 9, where the limbs are still relatively small and not always clearly visible (Figure 6A). On the other hand, using VR, segmentations with holes were observed (AI 0 cases versus VR 17 cases, Figure 6B). We found that both methods sometimes wrongly included parts of the umbilical cord or yolk sac (Figures 6C and 6D). For the HV, using AI, ears were more frequently missing (AI 5 cases, VR 1 case). For both AI and VR, segmentations were sometimes underestimating the boarder of the head (Figures 6D and 6E). Using AI, the cutting plane was more often estimated correctly (AI 2 cases, VR 6 cases, Figure 6F).

**Table 6:** Description of the minor and major adjustments denoted during the rating of both artificial intelligence and virtual reality segmentations, per gestational week for both raters.

				AI^a^		VR^b^
**EV^c^**						
	**Week 7**					
		Minor	Oversegmented: 3 Umbilical cord: 2 Yolk sac: 1		Undersegmented at border: 1 Holes: 7	
		Major	Limb missing: 8 Arm: 2 Leg: 6		Limb missing: 3 Arm: 2 Leg: 1	
	**Week 9**					
		Minor	Part of limb missing: 4 Hand: 1 Feed: 3 Oversegmented: 1 Umblical cord: 1		(Part of) limb missing: 1 Hand: 1 Oversegmented: 4 Umblical cord: 2 Yolk sac: 2 Holes: 4	
		Major	Limb missing: 10 Leg: 10		(Part of) limb missing: 4 Arm: 1 Leg: 3	
	**Week 11**					
		Minor	Part of limb missing: 5 Feed: 2 Hand: 3 Oversegmented: 1 Yolk sac: 1		Part of limb missing: 7 Leg: 2 Hand: 5 Holes: 6	
		Major	Limb missing: 5 Leg: 2 Arm: 3		Limb missing: 2 Leg: 2	
**HV^d^**						
	**Week 9**					
		Minor	Undersegmented at border: 3		Undersegmented at border: 5 Oversegmented: 3 Uterine wall: 1 Yolk sac: 2 Part of head missing: 1 Nose: 1 Holes: 7	
		Major	—^e^		Wrong cutting plane: 3	
	**Week 11**					
		Minor	Undersegmented at border: 1 Part of head missing: 5 Ears: 5		Oversegmented: 2 Hand: 1 Uterine wall: 1 Part of head missing: 1 Ears: 1 Holes: 13	
		Major	Wrong cutting plane: 2		Wrong cutting plane: 6	

^a^AI: artificial intelligence.

^b^VR: virtual reality.

^c^EV: embryonic volume.

^d^HV: head volume.

^e^Not applicable.

## Discussion

### Principal Findings

Volume measurements of the human embryo in the first trimester provide valuable insights into growth and development during this crucial period. Currently, obtaining these measurements is costly, laborious, and requires specialized training due to the in-house developed VR setup. Automating these measurements saves valuable time, and saves training and equipment costs. Eventually, this will enable the introduction of new, innovative volumetric parameters into clinical practice. To achieve automatic measurements, we proposed a state-of-the-art automatic AI algorithm, based on nnU-net [23], to segment the embryo and embryonic head, and subsequently measure the volume.

Quantitatively, we found that we could accurately measure the EV in gestational weeks 7 to 11 and the HV in weeks 9 to 11 GA on the test set, both with an ICC of 0.998 compared to measurements performed using VR. In addition, our method achieves a comparable ICC of 0.996 and 0.997 for EV and HV, respectively, when tested on images acquired in 2020. With an average combined measurement and rating time of less than 2 minutes, we can obtain accurate measurements of EV and HV in a time-efficient manner. Note that for EV and HV intrarater ICC is 0.999 and respectively >0.99, and interrater ICC is 0.999 and respectively >0.99, showing high reproducibility of VR measurements, which is comparable to reproducibility using AI [10,11].

We investigated the impact of GA, adverse outcomes, BMI, and image quality on the performance of the developed algorithm for EV measurements. We found that GA and image quality significantly impacted performance. For GA, we found that the EV measurements performed in gestational week 7 yielded significantly lower performance of the algorithm. This can be attributed to the size of the embryo at this stage, which makes volumetric measurements more challenging. In the case of adverse outcomes, this led to significantly lower performance. Given the small number of adverse outcomes in our dataset, we did not look into specific cases and how there were handled by the algorithm. Furthermore, we found that image quality significantly affected the algorithm’s performance for EV, with excellent quality leading to better performance and moderate quality leading to significantly worse performance. For HV, we investigated the influence of GA and BMI and found no significant differences. Given that the gestational age and image quality distribution between the test set and future test set were similar, this analysis does not explain the slight underestimation of the volume in the future test set.

Our qualitative analysis revealed important differences between the AI and VR segmentations. For the HV, the AI segmentations were rated as excellent in 79% of cases, compared with only 17% for VR. The main reason for this was the incorrect estimation of the cutting plane and holes in the VR segmentations. However, for the EV, we found that major errors were more common with the AI algorithm, as it more frequently missed limbs. In contrast, VR had relatively more minor errors, such as holes and under- or oversegmentation. It is important to note that any ultrasound images where segmentation of the limbs was not possible in VR were excluded. As a result, the VR measurements inherently lack cases with missing limbs, introducing a positive bias since major errors were excluded prior to the study. In addition, experienced raters were able to extrapolate missing limb positions in low-quality images, whereas the AI algorithm had difficulties correctly segmenting those cases. To address this challenge, an interesting direction of future research could be multiclass approaches, where rather than segmenting the embryo as one class, different parts of the body (eg, the head, trunk, and limbs) are segmented as separate classes, which allow the AI algorithm to explicitly learn about the shape and spatial ordering of these parts. Finally, the use of AI led to higher agreement among raters, possibly because major errors were more easily observable, especially at later gestational ages (weeks 9 and 11), or because the AI segmentations appeared smoother and simplified visual assessment.

The qualitative analysis was conducted using a fixed protocol to ensure consistent quality checking, with the time required for this process also recorded. Beyond evaluating our results qualitatively, we demonstrate how quality checking could be implemented in clinical practice and estimate the time it would require. Future work will aim to enhance this approach by incorporating failure-awareness into the automated measurement through the assessment of AI-model uncertainty [34].

Finally, we evaluated the precision of the AI measurements compared to the VR measurements. To this end, we analyzed the associations between birth weight and longitudinally measured CRL, EV obtained using VR, and EV obtained using AI. We found that the effect sizes (β) and 95% CIs were comparable for the EV measured using VR and AI. This finding shows that despite the difference in measurement method, growth trajectories were comparable estimated. This analysis showed no significant associations between birth weight and CRL or EV, however, studying associations was not the aim of this analysis due to the small sample size. For future work, we suggest that a more in-depth analysis to study the predictive value of EV compared with CRL should be performed using a larger sample size. Such an analysis should not only include the association with birth weight, but also the prediction of adverse outcomes such as miscarriage (associated with EV measured using VR [2]), congenital anomalies (associated with EV measured using VR [16]), small-for-gestational age, hypertensive disorders of pregnancy, and preterm birth.

### Comparison With Previous Work

In the literature, 3 comparable automatic AI methods for the segmentation of the embryo can be found [20-22]. All approaches used a U-net like architecture for EV segmentation, Looney et al [20] and Yang et al [22] combined this both with transfer learning and either label refinement, or a 2-pathway architecture. Ryou et al [21] subsequently segmented the HV by using a multitask approach. Looney et al [20] achieved a mean Dice score of 0.876 for the EV (tested on 60 3D volumes, 10-14 weeks GA using a GE Voluson 730 expert system), Yang et al [22] achieved 0.880 for the EV (tested on 44 volumes, 10-14 weeks GA using a Mindray DC-8 machine with a 3D 23.8-8.2 MHz probe), and Ryou et al [21] achieved for the EV a mean accuracy of 89.4% and for the HV 95.4% (tested on 21 3D volumes, 11-14 weeks GA using a Philips HD9 machine with a 3D V7-3 probe) [20]. In contrast, our AI algorithm, tested on hundreds of images, can measure EV from gestational week 7 and HV from gestational week 9, until week 11*.*

### Limitations

A limitation of our model is that it was developed and tested only on imaging data acquired within the same institute, using one type of ultrasound system. Evaluation in other settings is needed to investigate generalizability. Currently, only datasets containing 2D scans of standard planes are publicly available, which are not suitable for volume segmentation [35,36]. To expand the applicability of our model in different clinical settings, training and validation on an extended dataset containing data from multiple sources is needed. However, for development, segmentations are needed, which are not always available, and are time-consuming and costly to obtain. To investigate the influence of having fewer segmentations available, we subdivided the development dataset into smaller partial development datasets. We found that for smaller development datasets the spread in the performance gets wider, but decreases only slightly for the Dice score. We found that the smallest dataset, which used contained 18 (EV) and 21 images (HV), still achieved mean Dice scores of 0.888 and 0.946 respectively, compared with 0.935 and 0.955 on the full dataset. This suggests that accurate results can still be achieved even with a limited number of segmentations available. This opens up opportunities for further research, such as exploring the use of a similar approach for measuring the yolk sac, gestational sac, brain ventricles, and the utero placental (vascular) volume. All these measurements have been performed before within the Rotterdam Periconception cohort, but to date only limited or no segmentations are available [9,37].

A possible drawback of our study is that the second and third rater in the qualitative experiments had no experience in performing ultrasonography. However, the 3D VR visualization of the ultrasound image simplifies the assessment of the image, which reduces the need for experience in ultrasonography. This is supported by the agreement with the first rater (gynecologist, >10 years of experience in VR measurements), which was substantial. Moreover, both the second and third rater followed standardized training to perform these measurements [28].

Although EV provides a more accurate estimation of embryonic growth than CRL, CRL remains the clinical gold standard [8,12]. A limitation of our study is that we focused on automating a novel measurement rather than the clinical standard. However, performing automatic biometric measurements like CRL or head circumference from 3D volumes requires the identification of corresponding standard planes. While EV and HV segmentation could simplify some aspects of this process, they do not directly address the plane detection needed for these measurements. Automatic standard plane detection has been successfully applied to 2D ultrasonography, primarily in the second and third trimesters [36,38]. We have conducted preliminary work on extracting the midsagittal plane via spatial alignment for CRL measurement, which yielded promising results [39].

### Conclusions

We showed that we can accurately measure the EV in gestational weeks 7 to 11 and the HV in gestational weeks 9 to 11 using a fully automatic algorithm. We achieve this in a time-efficient manner, saving significant time over current approaches. The strength of our study was the combination of using a state-of-the-art AI algorithm for medical image segmentation, the availability of a large dataset, and thorough quantitative and qualitative evaluation. Especially, the expert ratings revealed important differences between AI and VR measurement. We showed that beside being reliable, performing measurements using AI is time-saving, even when taking the subsequent quality checking into account. Ultimately, automating volumetric measurements may save valuable time in clinical practice, as a screening and diagnostic tool early in pregnancy for adverse pregnancy outcomes, fetal growth restriction, and congenital anomalies. Early diagnosis leaves room for treatment and interventions leading to better pregnancy outcomes and the lifelong health of the developing embryo and fetus. Moreover, besides measuring the volume, segmentations provide important spatial and morphological information that is crucial for other image analysis tasks such as standard plane detection, anomaly detection, and the development of data-driven spatiotemporal growth models [39,40].

## Appendix

Multimedia Appendix 1 Technical details nnU-net.

Multimedia Appendix 2 Data characteristics of subsampled development datasets.

Multimedia Appendix 3 Additional tables and figures of the association study.

Multimedia Appendix 4 Qualitative analysis subgroup characteristics.

Multimedia Appendix 5 Intrarater agreement qualitative rating.

## Abbreviations

AI: artificial intelligence
CRL: crown-rump length
EV: embryonic volume
GA: gestational age
HV: head volume
ICC: intraclass correlation coefficient
ICSI: intracytoplasmic sperm injection
IVF: in vitro fertilization
VOCAL: virtual organ computer added analysis
VR: virtual reality

## Data Availability

The datasets generated or analyzed during this study are not publicly available to privacy regulations but are available from the corresponding author on reasonable request.
