# Supplementary material for: Automatic Human Embryo Volume Measurement in First Trimester Ultrasound From the Rotterdam Periconception Cohort: Quantitative and Qualitative Evaluation of Artificial Intelligence
Source: J Med Internet Res. 2025 Mar 31;27:e60887. doi: 10.2196/60887 (PMC11997536; doi:10.2196/60887)
Supplement: Multimedia Appendix 1 [file jmir_v27i1e60887_app1.docx]

**Supporting information 1: technical details nnU-net**

**Choice of model**nnU-net encompasses three different models, 2D U-Net, 3D U-net and U-Net cascade, which can be used independently or in an ensemble, depending on the dataset and computational resources at hand (1). U-net is a type of neural network architecture that is used for segmentation (2). Here, only the 3D U-net model is used. The 2D model is not applicable since this is only advised in the case of anisotropic voxel spacing, which is not the case for the ultrasound data used here. The available computational resources, in combination with the size of the images used, determine the size of the subregions extracted from the images. If these subregions are sufficiently small, the cascade model is advised, however, in our case the subregions were too large.

**Training of the model**Both models, for the embryonic volume (EV) and head volume (HV), were trained using the default hyperparameters using 5-fold cross-validation. We trained nnU-net version 1.6.5 on a Nvidia A40 48GB GPU with an AMD EPYC 7742 CPU using 20 GB of RAM.

**Inferred hyperparameters**nnU-net infers the following hyperparameters based on the data used for training:

- Target spacing: spacing to which all image are resampled.
- Patch size: large patch size is chosen over large batch size.
- Batch size.
- Median image size after resampling.
- Number of down-sampling operations per axis: is chosen such that the feature map is reduced to 4 voxels.
- Kernel size convolutions: default for the 3D model is 3x3x3.

In **Table S1.1** the hyperparameters inferred for the embryonic volume (EV) are given, and in **Table S1.2** the ones for the head volume (HV) model.

**Table S1.1** Inferred hyperparameters of the embryonic volume (EV) model.

|  | Hyperparameter | Value |
| --- | --- | --- |
| Resampling |  |  |
|  | Target spacing | [0·21, 0·21, 0·21] |
| Network topology |  |  |
|  | Patch size | [160, 112, 128] |
|  | Batch size | 2 |
|  | Median image size after resampling | [225, 156, 221] |
|  | Number of down-sampling operations per axis | [5, 4, 5] |
|  | Kernel size convolutions | 3x3x3 |

**Table S1.2** Inferred hyperparameters of the head volume (HV) model.

|  | Hyperparameter | Value |
| --- | --- | --- |
| Resampling |  |  |
|  | Target spacing | [0·23, 0·23, 0·23] |
| Network topology |  |  |
|  | Patch size | [160, 96, 160] |
|  | Batch size | 2 |
|  | Median image size after resampling | [216, 151, 225] |
|  | Number of down-sampling operations per axis | [5,4,5] |
|  | Kernel size convolutions | 3x3x3 |
