# Supplementary material for: Automatic Human Embryo Volume Measurement in First Trimester Ultrasound From the Rotterdam Periconception Cohort: Quantitative and Qualitative Evaluation of Artificial Intelligence
Source: J Med Internet Res. 2025 Mar 31;27:e60887. doi: 10.2196/60887 (PMC11997536; doi:10.2196/60887)
Supplement: Multimedia Appendix 2 [file jmir_v27i1e60887_app2.docx]

**Supporting information 2: data characteristics of subsampled development datasets**

**Table S2.1 Data characteristics of the subsampled development datasets for the embryonic and head volume measurement.**

|  | 100% | 80% | 60% | 40% | 20% | 10% | 5% | 2·5% |
| --- | --- | --- | --- | --- | --- | --- | --- | --- |
| EV |  |  |  |  |  |  |  |  |
| Number of patients | 341 | 273 | 205 | 136 | 68 | 34 | 17 | 9 |
| Number of ultrasound scans | 648 | 511 | 380 | 268 | 137 | 67 | 33 | 18 |
| GA |  |  |  |  |  |  |  |  |
| Week 7 | 21·8% | 21·3% | 20·5% | 23·5% | 23·4% | 23·9% | 27·3% | 38·9% |
| Week 9 | 38·3% | 38·4% | 39·5% | 36·6% | 40·0% | 35·8% | 33·3% | 27·8% |
| Week 11 | 28·4% | 28·0% | 28·2% | 28·7% | 29·9% | 29·9% | 27·3% | 27·8% |
| Other | 11·5% | 12·3% | 11·8% | 11·2% | 6·7% | 10·4% | 12·1% | 5·5% |
| BMI |  |  |  |  |  |  |  |  |
| Not obese (<30) | 85·4% | 85·1% | 84·8% | 86·2% | 86·1% | 85·1% | 81·8% | 94·4% |
| Obese (≥30) | 14·4% | 14·5% | 14·7% | 13·8% | 13·9% | 14·9% | 18·2% | 5·6% |
| Missing | 0·2% | 0·4% | 0·5% | 0·0% | 0·0% | 0·0% | 0·0% | 0·0% |
| Ultrasound quality |  |  |  |  |  |  |  |  |
| Excellent | 30·9% | 30·3% | 29·5% | 32·8% | 32·8% | 31·3% | 39·4% | 27·8% |
| Good | 39·2% | 41·2% | 41·3% | 36·2% | 31·4% | 31·3% | 42·2% | 16·7% |
| moderate | 27·6% | 25·8% | 26·6% | 29·1% | 34·3% | 35·8% | 18·2% | 55·5% |
| Missing | 2·3% | 2·7% | 2·6% | 1·9% | 1·5% | 1·6% | 0·0% | 0·0% |
| HV |  |  |  |  |  |  |  |  |
| Number of patients | 261 | 209 | 157 | 104 | 52 | 26 | 13 |  |
| Number of ultrasound scans | 383 | 302 | 224 | 159 | 81 | 40 | 21 |  |
| GA |  |  |  |  |  |  |  |  |
| Week 9 | 48·3% | 48·3% | 48·2% | 48·4% | 48·1% | 52·5% | 57·1% |  |
| Week 11 | 45·7% | 45·7% | 44·6% | 47·2% | 45·7% | 45·0% | 42·9% |  |
| Other | 6·0% | 6·0% | 7·1% | 4·4% | 6·3% | 2·5% | 0·0% |  |
| BMI |  |  |  |  |  |  |  |  |
| Not obese (<30) | 85·1% | 84·8% | 84·4% | 86·1% | 86·4% | 85·0% | 85·7% |  |
| Obese (≥30) | 14·9% | 15·2% | 15·6% | 13·9% | 13·6% | 15·0% | 14·3% |  |
| Missing | 0·0% | 0·0% | 0·0% | 0·0% | 0·0% | 0·0% | 0·0% |  |
