# Supplementary material for: Automatic Human Embryo Volume Measurement in First Trimester Ultrasound From the Rotterdam Periconception Cohort: Quantitative and Qualitative Evaluation of Artificial Intelligence
Source: J Med Internet Res. 2025 Mar 31;27:e60887. doi: 10.2196/60887 (PMC11997536; doi:10.2196/60887)
Supplement: Multimedia Appendix 3 [file jmir_v27i1e60887_app3.docx]

**Supporting information 3:** **Additional tables and figures of the association study**

**Figure S3.1 Scatterplot of the relationship GA and EV after log transformation.**

**
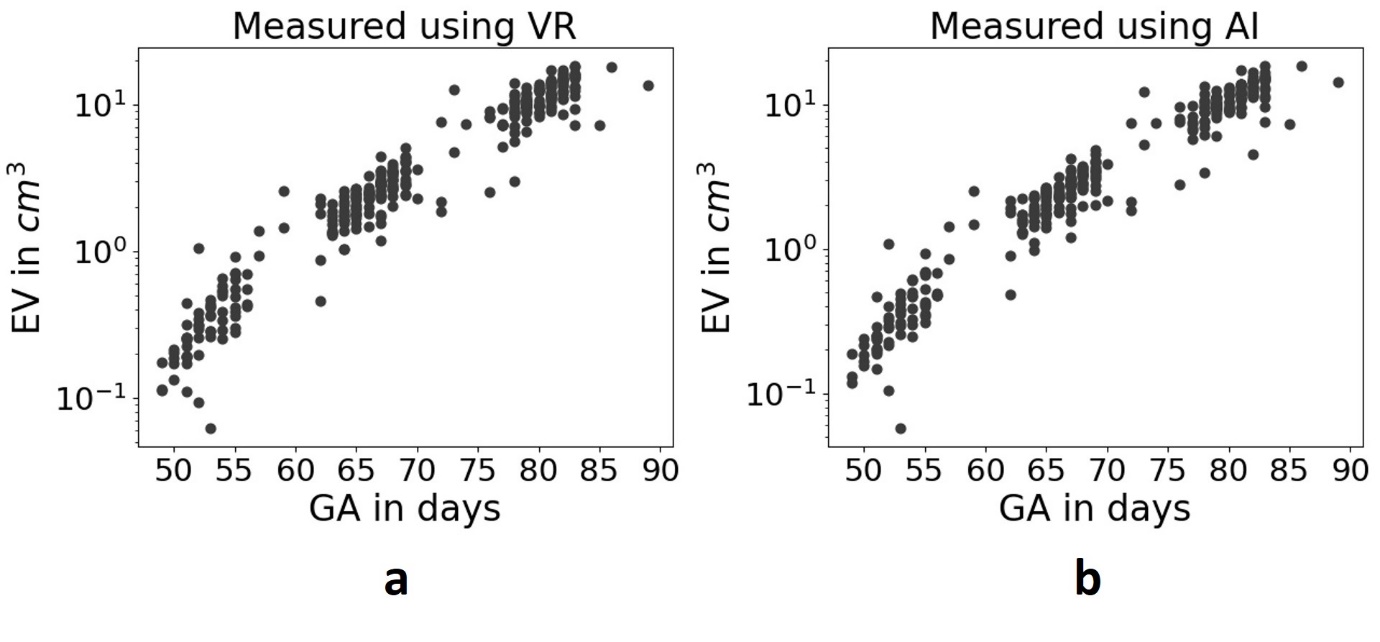
**

***a)*** *EV measured using VR.* ***b)*** *EV measured using AI.*

**Figure S3.2 Flowchart of study population used in the association study.**


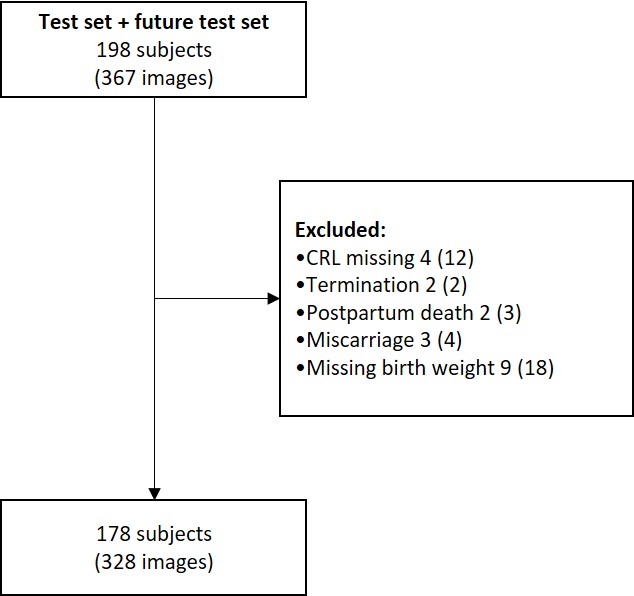


*CRL: crown-rump length.*

**Table S3.1 Data characteristics of the study population to analyse the relationship birthweight and CRL, EV measured using VR, and EV measured using AI.**

|  | Study population |
| --- | --- |
| Number of subjects (n) | 178 |
| Number of ultrasound scans (n) | 328 |
| GA (days) | 68·8 (10·4) |
| CRL (mm) |  |
| Week 7 | 13·0 (2·6) |
| Week 9 | 26·6 (3·2) |
| Week 11 | 49·5 (5·3) |
| Birth weight (g) | 3307 (590) |

*Standard deviation is given between brackets.*
