# Supplementary material for: Automatic Human Embryo Volume Measurement in First Trimester Ultrasound From the Rotterdam Periconception Cohort: Quantitative and Qualitative Evaluation of Artificial Intelligence
Source: J Med Internet Res. 2025 Mar 31;27:e60887. doi: 10.2196/60887 (PMC11997536; doi:10.2196/60887)
Supplement: Multimedia Appendix 4 [file jmir_v27i1e60887_app4.docx]

**Supporting information 4:** **Qualitative analysis subgroup characteristics**

**Table S4.1 Subgroup characteristics EV.**

| GA | Excellent | Good | Moderate | Not obese | obese | No adverse outcome | Adverse outcome |  | Total |
| --- | --- | --- | --- | --- | --- | --- | --- | --- | --- |
| Week 7 | 16 (40.0%) | 11 (27.5%) | 16 (40.0%) | 34 (85.0%) | 6  (15.0%) | 37 (92.5%) | 3  (7.5%) |  | 40 (100.0%) |
| Week 9 | 27 (40.3%) | 22 (32.8%) | 16 (23.9%) | 55 (82.1%) | 12 (17.9%) | 61 (91.0%) | 6  (9.0%) |  | 67 (100.0%) |
| Week 11 | 16  (36.4%) | 14  (31.8%) | 14 (31.8%) | 39  (88.6%) | 5  (11.4%) | 43  (97.7%) | 1  (2.3%) |  | 44 (100.0%) |
| Outcome | **Week 7** | **Week 9** | **Week 11** | **Excellent** | **Good** | **Moderate** | **Not obese** | **Obese** |  |
| No adverse outcome | 37  (24.5%) | 61  (40.4%) | 43  (28.5%) | 55  (36.4%) | 50  (33.1%) | 43  (28.5%) | 130  (86.1%) | 21  (13.9%) | 151 (100.0%) |
| Adverse outcome | 3  (25.0%) | 6  (50.0%) | 1  (8.3%) | 4  (33.3%) | 3  (25.0%) | 5  (41.7%) | 10  (83.3%) | 2  (16.7%) | 12 (100.0%) |
| Obesity | **Week 7** | **Week 9** | **Week 11** | **Excellent** | **Good** | **Moderate** | **No adverse outcome** | **Adverse outcome** |  |
| Not obese | 34  (24.1%) | 55  (39.0%) | 39  (27.7%) | 53  (37.6%) | 44  (31.2%) | 41  (29.1%) | 130  (92.9%) | 10  (7.1%) | 141 (100.0%) |
| Obese | 6  (26.1%) | 12  (52.2%) | 5  (21.7%) | 6  (26.1%) | 9  (39.1%) | 7  (30.4%) | 10  (43.5%) | 2  (8.7%) | 23 (100.0%) |
| Image quality | **Week 7** | **Week 9** | **Week 11** | **Not obese** | **obese** | **No adverse outcome** | **Adverse outcome** |  |  |
| Excellent | 16  (27.1%) | 27  (45.8%) | 16  (27.1%) | 53  (89.8%) | 5  (8.5%) | 55  (93.2%) | 4  (6.8%) |  | 59 (100.0%) |
| Good | 11  (20.8%) | 22  (41.5%) | 14  (26.4%) | 44  (83.0%) | 6  (11.3%) | 50  (94.3%) | 3  (5.7%) |  | 53 (100.0%) |
| Moderate | 16  (32.7%) | 16  (32.7%) | 14  (28.6%) | 41  (83.7%) | 9  (18.4%) | 43  (87.8%) | 5  (10.2%) |  | 49 (100.0%) |

**Table S4.2 Subgroup characteristics HV**

| GA | Not obese | obese | Total |
| --- | --- | --- | --- |
| Week 9 | 36  (83.7%) | 7  (16.3%) | 43 (100.0%) |
| Week 11 | 37  (84.1%) | 7  (15.9%) | 44  (100.0%) |
| Obesity | **Week 9** | **Week 11** |  |
| Not obese | 36  (46.2%) | 37  (47.4%) | 78  (100.0%) |
| Obese | 7  (50.0%) | 7  (50.0%) | 14  (100.0%) |
