# Supplementary material for: Automatic Human Embryo Volume Measurement in First Trimester Ultrasound From the Rotterdam Periconception Cohort: Quantitative and Qualitative Evaluation of Artificial Intelligence
Source: J Med Internet Res. 2025 Mar 31;27:e60887. doi: 10.2196/60887 (PMC11997536; doi:10.2196/60887)
Supplement: Multimedia Appendix 5 [file jmir_v27i1e60887_app5.docx]

**Supporting information 4: Intra-rater agreement qualitative rating**

**
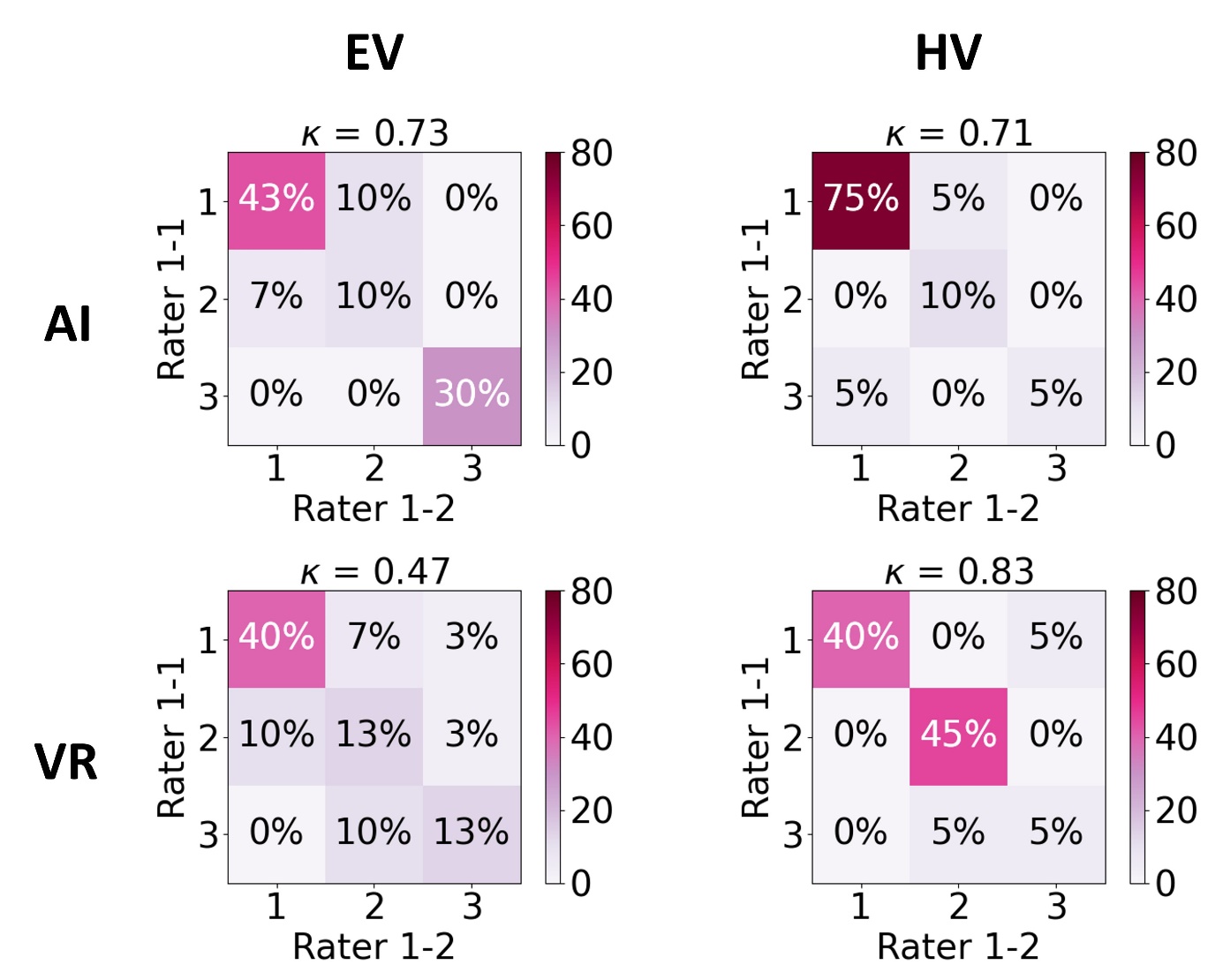
**

**Figure 5: Confusion matrices for the qualitative rating, showing the scores given by the two repetitions of round A by rater 1.** Rater 1-1 refers to rating given by rater 1 during the first repetition, rater 1-2 refers to the rating given during the second repetition. The percentages on the diagonal show where the rater agreed. Score 1 indicates that no adjustments were needed, score 2 indicate that only minor adjustments were needed (filling holes, adding part of limb, under- or over-segmentation at the border), and score 3 indicates that major adjustments were needed (missing limbs, wrongly estimated cutting plane). Cohen’s kappa is indicated by κ, where κ between 0.21 to 0.4 indicates fair agreement and κ between 0.61 to 0.8 substantial agreement.
